# Members of the GADD45 Protein Family Show Distinct Propensities to form Toxic Amyloid-Like Aggregates in Physiological Conditions

**DOI:** 10.3390/ijms221910700

**Published:** 2021-10-02

**Authors:** Giovanni Smaldone, Daniela Caruso, Annamaria Sandomenico, Emanuela Iaccarino, Annalia Focà, Alessia Ruggiero, Menotti Ruvo, Luigi Vitagliano

**Affiliations:** 1 IRCCS SDN, Napoli, Via E. Gianturco 113, 80143 Napoli, Italy; giovanni.smaldone@synlab.it; 2 Institute of Biostructures and Bioimaging, C.N.R., Via Mezzocannone 16, 80134 Napoli, Italy; dan.caruso@virgilio.it (D.C.); annamaria.sandomenico@cnr.it (A.S.); emanuela.iaccarino@gmail.com (E.I.); annalia.foca@gmail.com (A.F.); alessia.ruggiero@cnr.it (A.R.)

**Keywords:** protein aggregation, amyloid-like toxicity, structure-stability relationships

## Abstract

The three members (GADD45α, GADD45β, and GADD45γ) of the growth arrest and DNA damage-inducible 45 (GADD45) protein family are involved in a myriad of diversified cellular functions. With the aim of unravelling analogies and differences, we performed comparative biochemical and biophysical analyses on the three proteins. The characterization and quantification of their binding to the MKK7 kinase, a validated functional partner of GADD45β, indicate that GADD45α and GADD45γ are strong interactors of the kinase. Despite their remarkable sequence similarity, the three proteins present rather distinct biophysical properties. Indeed, while GADD45β and GADD45γ are marginally stable at physiological temperatures, GADD45α presents the Tm value expected for a protein isolated from a mesophilic organism. Surprisingly, GADD45α and GADD45β, when heated, form high-molecular weight species that exhibit features (ThT binding and intrinsic label-free UV/visible fluorescence) proper of amyloid-like aggregates. Cell viability studies demonstrate that they are endowed with a remarkable toxicity against SHSY-5Y and HepG2 cells. The very uncommon property of GADD45β to form cytotoxic species in near-physiological conditions represents a puzzling finding with potential functional implications. Finally, the low stability and/or the propensity to form toxic species of GADD45 proteins constitute important features that should be considered in interpreting their many functions.

## 1. Introduction

The growth arrest and DNA damage-inducible 45 (GADD45) gene family encodes three strictly related proteins, denoted as GADD45α, GADD45β, and GADD45γ [1,2,3,4,5]. Although the three members of the GADD45 protein family share quite similar sequences, with identities falling in the range 55–57%, they play distinct, albeit generally crucial roles in cell life. Indeed, literature surveys indicate that GADD45 proteins are implicated in a countless number of physio–pathological processes [6]. These include DNA repair, cell cycle control, senescence and genotoxic stress, and tumorigenesis. GADD45 proteins accomplish these diversified functions by interacting with a multitude of biological partners [4]. The most important functions of GADD45α, the best-characterized member of the family, are related to the induction of growth arrest and DNA repair, indicating a crucial role in maintaining genomic stability, in the DNA damage response, and in cancer transformation. Although direct data on the biochemical mechanisms underlying GADD45α functions are still poor, this protein mostly operates by promoting protein–protein interactions or by sequestering specific partners. These include p53, cyclin dependent kinase 1 (CDK1), and cyclin B1. It is also important to note that the basal expression GADD45α is very low, and that it is regulated by a multitude of external factors that include both physical and biochemical signals, such as ultra violet irradiation, X-rays, γ-irradiation, and DNA toxins. Although GADD45β and GADD45γ have not been extensively characterized yet, several reports have highlighted their important functional roles. Recent studies have shown that GADD45β plays an important role in multiple myeloma onset and maintenance. Extensive cellular and molecular investigations have demonstrated that this protein suppresses the pro-apoptotic JNK signaling through a direct inhibition of the upstream kinase mitogen-activated protein kinase 7 (MKK7) in this type of cancer [7]. Since the GADD45β/MKK7 complex is critical for the NF-κB-driven survival, it is a promising therapeutic target in multiple myeloma [8,9]. Moreover, GADD45β was recently associated with colorectal carcinoma, and could be used as a prognostic and predictive biomarker of the disease [10]. GADD45γ, which is the most evolutionary conserved protein in the family, is clearly defined as a pro-apoptotic and cell cycle arrest–inducing protein. Very recently, it was shown that this protein is selectively silenced in acute myeloid leukemia, thus providing insight into the design of novel therapeutic strategies used to combat this disease [11]. The involvement of the members of the GADD45 family in diversified biological processes, despite their overall sequence similarity, prompted us to undertake further biochemical and biophysical studies in order to highlight analogies and differences. In this framework, we assessed the ability of all GADD45 members in specifically binding the MKK7 kinase. Moreover, the thermal stability and the propensity of GADD45α, GADD45β, and GADD45γ to form amyloid-like cytotoxic aggregates have been unraveled.

## 2. Results

### 2.1. Production of the Recombinant Proteins and Initial Biophysical Characterizations

Recombinant GADD45α, GADD45β, and GADD45γ were obtained in amounts sufficient for performing all subsequent characterizations. The proteins were initially purified by affinity chromatography exploiting the presence of the histidine tag located at the N-terminus. SEC was used to remove aggregates or residual contaminants. All proteins had purities higher than 95% as estimated from by SDS–PAGE analysis (Figure S1). The molecular weights of the three recombinant proteins were in close agreement with the calculated values (Table S1).

**Figure 1:**
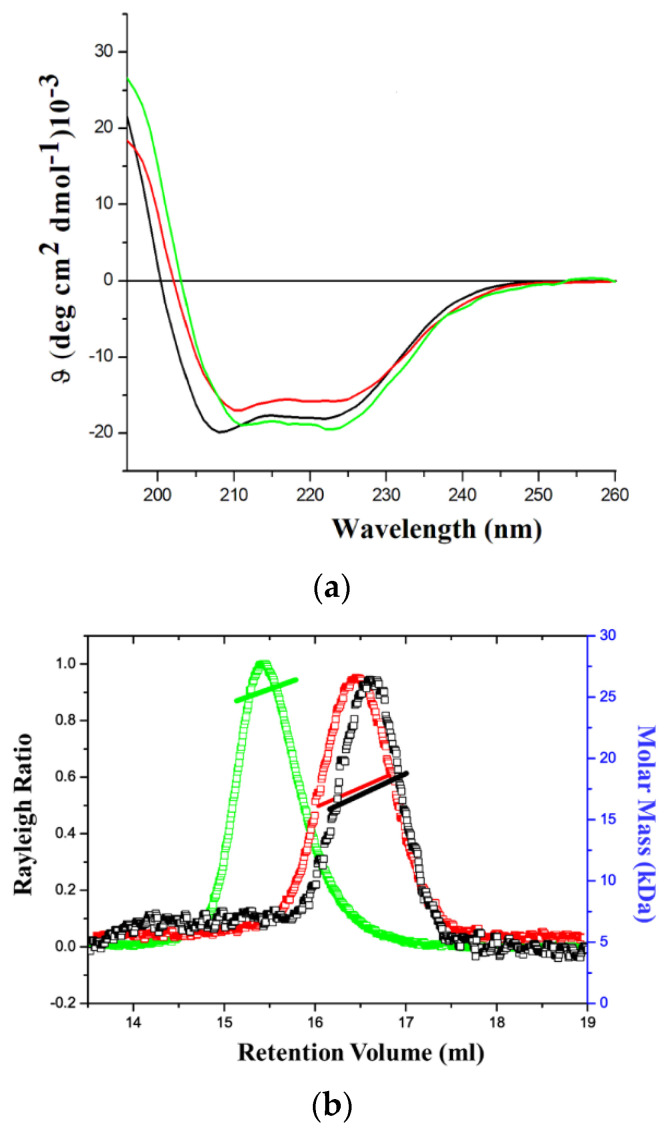
Far−UV CD spectra recorded at 20 °C of GADD45α (black line), GADD45β (red line), and GADD45γ (green line) (**a**). (**b**) SEC-LS analysis of GADD45α (black line), GADD45β (red line), and GADD45γ (green line). The SEC-LS experiments were performed using a Superdex S200 10/30 column pre-equilibrated with a buffer containing 5 mM DTT in 20 mM Tris-HCl buffer—pH 7.5.

In order to assess their folding state, the three proteins were analyzed by far-UV CD spectroscopy. The resulting CD spectra of GADD45α, GADD45β, and GADD45γ clearly indicate that all of them were well folded (Figure 1a). Indeed, the occurrence in the spectra of a maximum (at about ~198 nm) and of two minima (at ~208 nm and ~222 nm) suggest that the three isoforms contain both α- and β-structure elements [12,13,14] as expected on the basis of the reported three-dimensional structures of GADD45α [15] and GADD45γ [16,17], and of the sequence similarities among the different isoforms (Figure S2). The oligomeric state of GADD45α, GADD45β, and GADD45γ was evaluated by static light scattering analyses. As shown in Figure 1b, in the experimental conditions (5 mM DTT in 20 mM Tris-HCl buffer—pH 7.5), GADD45α and GADD45β are monomeric, while GADD45γ presents a dimeric organization.

### 2.2. Binding of GADD45 Proteins to MKK7

**Figure 2:**
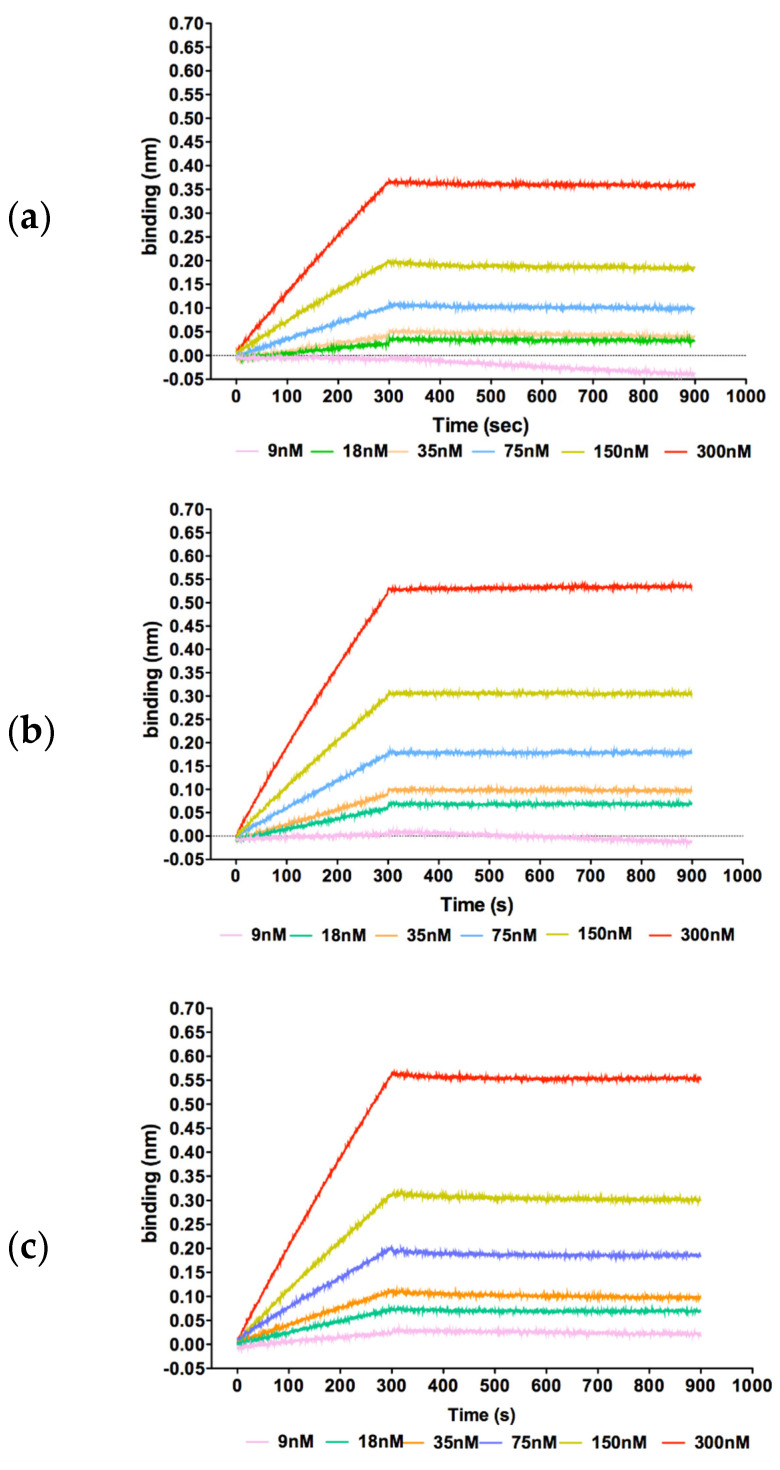
Sensorgrams showing the binding between the kinase domain of MKK7 and the immobilized GADD45α (**a**), GADD45β (**b**), and GADD45γ (**c**). Lines represent different concentrations, whose color code is reported under the graph, of MKK_KD protein.

Once assessed that recombinant GADD45α, GADD45β, and GADD45γ were properly folded, we comparatively evaluated their ability to bind the kinase domain of MKK7, so far described only for GADD45β [7,9,18]. Binding of the three isoforms to MKK7_KD was investigated by Bio-Layer Interferometry. The approach was initially validated quantifying the GADD45β-MKK7_KD affinity that was previously determined by surface plasmon resonance (SPR). We found that GADD45β bound MKK7_KD with a KD of 2.0 × 10^−9^ M, a value in line with the one previously determined by SPR (6.0 × 10^−9^ M) [9] (Figure 2b). Interestingly, we also found that the other two GADD45 isoforms bound the kinase domain in a dose-dependent manner (Figure 2a,c). In particular, the KD values of the complexes GADD45α/MKK7_KD and GADD45γ/MKK7_KD were 2.3 × 10^−8^ and 1.5 × 10^−8^, respectively. These values indicate a significant reduced affinity of this isoforms for MKK7_KD compared to GADD45β. These findings indicate that all GADD45 isoforms share the ability to bind the MKK7 kinase domain, although GADD45α and GADD45γ present a 10-fold reduced affinity compared to GADD45β.

### 2.3. Thermal Stability of GADD45 Proteins

**Figure 3:**
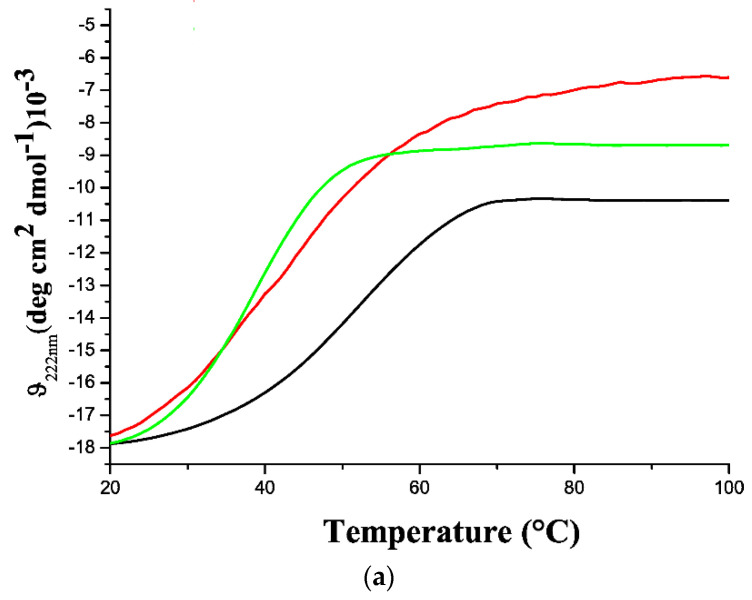

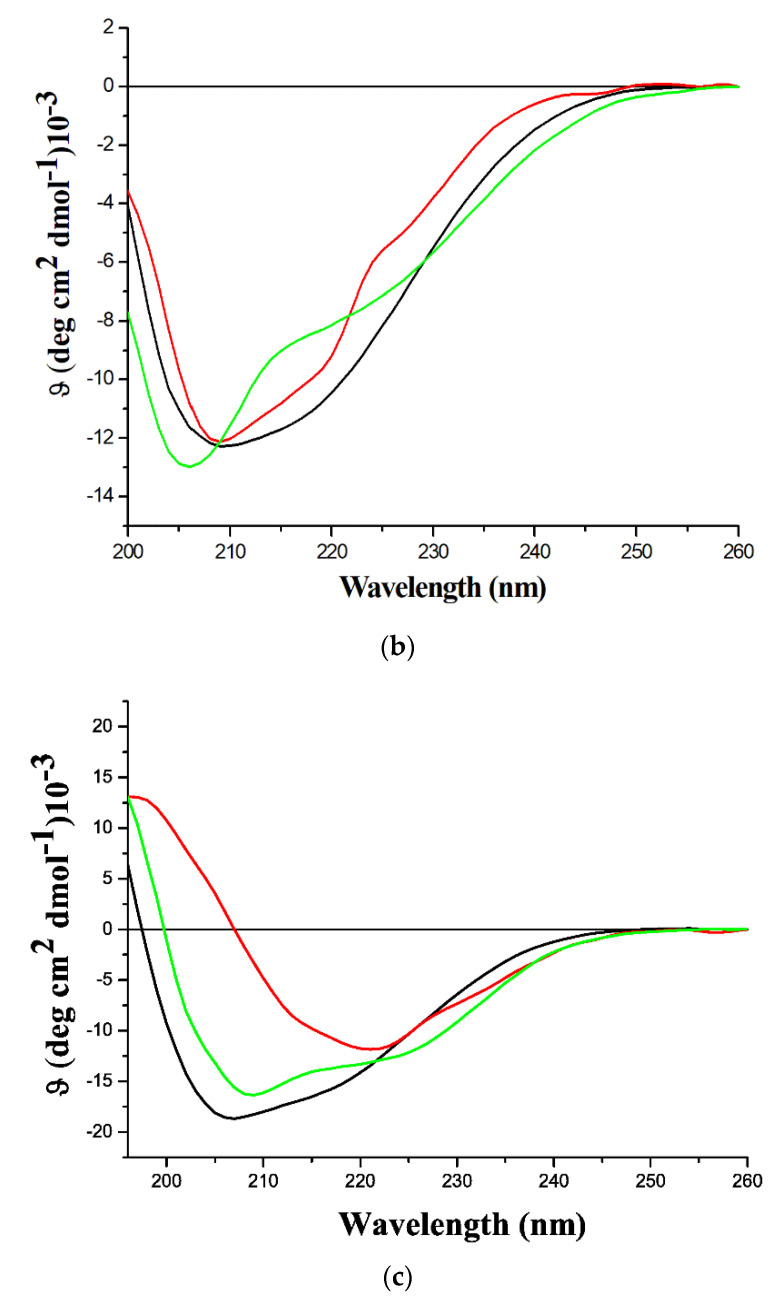
(**a**) Thermal denaturation curves of GADD45α (black line), GADD45β (red line), and GADD45γ (green line). Far-UV CD spectra recorded at 100 °C (**b**) and 20 °C (**c**) after thermal denaturation of GADD45α (black line), GADD45β (red line), and GADD45γ (green line). Experiments are representative of three independent measurements with similar results.

The analysis of the stability against temperature was performed by a CD signal at 222 nm in the temperature range 20–100 °C. In all cases, structural transitions were observed at temperatures much lower than 100 °C. Moreover, the strong CD signals detected after the transition indicates that all proteins remained soluble in the explored temperature interval. Despite these analogies, the thermal denaturation experiments highlighted significant differences among GADD45 proteins (Figure 3a). Indeed, the monitoring of the CD signal indicated that GADD45α is a rather stable protein with a melting temperature Tm of 53 °C. On the other hand, GADD45β and GADD45γ are barely stable at temperatures close to the physiological ones as they both present Tm values of around 40 °C. A deep inspection of the melting curves indicated that the CD signal of GADD45β and GADD45γ presented significant variations from the starting value, even at rather low temperatures (~30 °C) (Figure 3b). This is particularly evident for GADD45β, whose unfolding is essentially non-cooperative. These observations indicate that the folded state of GADD45β is somewhat heat-labile and prone to undergo structural transitions. 

Interestingly, the analysis of the structural properties of GADD45α, GADD45β, and GADD45γ states obtained upon temperature treatment unravels peculiar and sequence-specific behaviors. As expected, the CD spectra of the three proteins recorded at 100 °C (Figure 3b) are radically different from those collected at room temperature (Figure 1a). The CD spectrum of GADD45γ presents a minimum at 205 nm and a shoulder at 220 nm that are indicative of a denatured protein with a non-negligible portion of residual secondary structure. When GADD45γ samples are cooled back at room temperature, the protein virtually recovers its native structure (Figure 3c). A rather different scenario emerges from the characterization of the thermally treated samples of GADD45α and GADD45β. For these isoforms, the secondary structure undergoes major variations upon heating (Figure 1a and Figure 3b). In particular, the CD spectra collected at 100 °C present a unique minimum located at about 210 nm, indicative of β-rich structures, and their overall appearance is preserved when samples are cooled at room temperature (Figure 3c).

### 2.4. Characterization of the β-Rich States Formed by GADD45α/GADD45β

**Figure 4:**
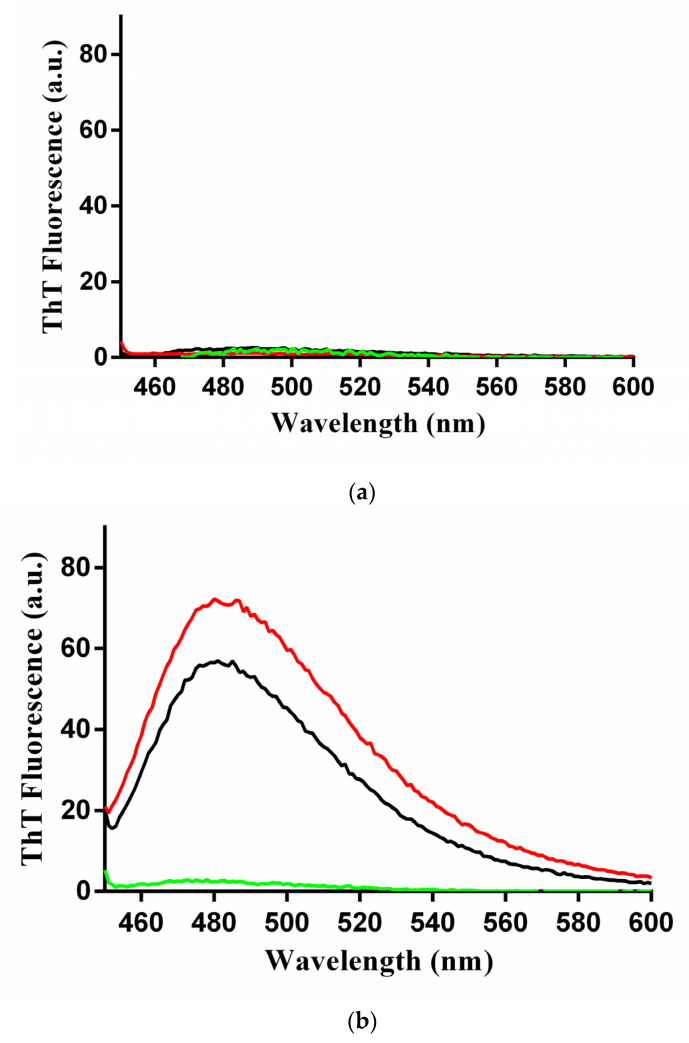

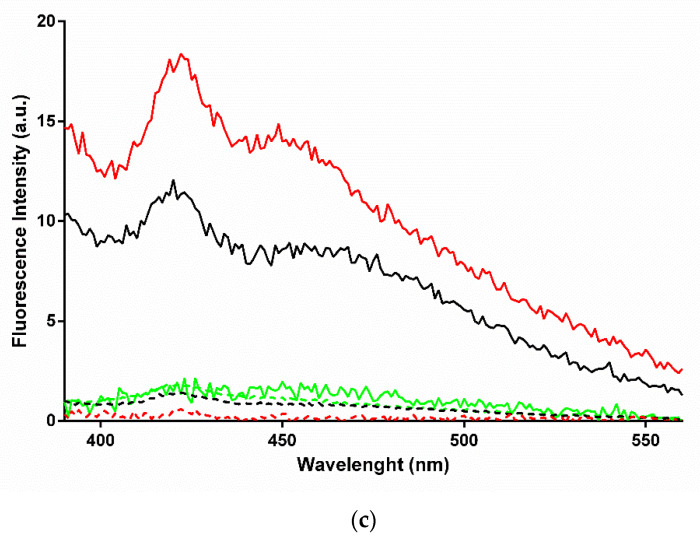
ThT fluorescence spectra of thermally treated GADD45α (black line), GADD45β (red line), and GADD45γ (green line), (**a**) and native GADD45α (black line), GADD45β (red line), and GADD45γ (green line) (**b**). Fluorescence was reported as Arbitrary Unit after excitation at 440 nm and recorded between 450 and 600 nm. (**c**) Fluorescence Intensity, after excitation at 370 nm, of native (dotted lines) and thermal treated (continuous lines) GADD45α, GADD45β, and GADD45γ. Spectra were recorded between 390 and 560 nm. Experiments are representative of three independent measurements with similar results.

**Figure 5:**
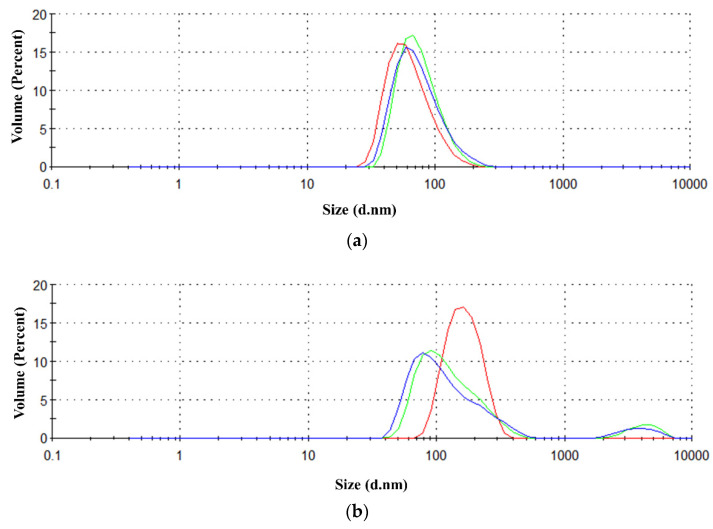
Size distribution by volume of GADD45α (**a**) and GADD45β (**b**) after thermal denaturation, obtained by dynamic light scattering measurements. The curves represent three independent measurements. Size is reported as diameter of protein tested, in nanometer.

A number of experiments were designed and performed to investigate the structural features of GADD45α/GADD45β states generated by the thermal treatment. To gain insights into the structural features of the β-rich aggregates formed by GADD45α and GADD45β, we evaluated their ability to interact with ThT, a probe widely used to assess the formation of amyloid-like assemblies [19]. ThT emits a specific fluorescence at ~480 nm when excited at ~440 nm upon binding to these structures. As shown in Figure 4a, no ThT fluorescence was detected for the three isoforms in their native state. Interestingly, the thermally treated samples of GADD45α and GADD45β presented a strong ThT fluorescence signal with a maximum at 485 nm (Figure 4b). On the contrary, the thermally treated sample of GADD45γ, which does not form β-rich aggregates after denaturation, did not show ThT fluorescence emission. These findings suggested that, upon heating, GADD45α and GADD45β form amyloid-like assemblies. To corroborate this observation, we also evaluated the ability of these assemblies to emit the intrinsic blue fluorescence exhibited by amyloid-like assemblies. As shown in Figure 4c, none of the three native GADD45 isoforms presented the intrinsic blue fluorescence emission when excited at 370 nm. In line with the ThT experiments, only the thermally treated samples of GADD45α and GADD45β presented the emission of fluorescence with a maximum at 422 nm and a shoulder at about 455 nm. Finally, dynamic light scattering (DLS) experiments clearly indicated that, upon heating, both GADD45α and GADD45β were able to form assemblies with large hydrodynamic diameters (Figure 5). Indeed, the assemblies formed by GADD45α and GADD45β exhibited a diameter of 35 ± 3 and 86 ± 11 nm, respectively. Similar results were obtained following the size distribution either by volume (Figure 5) or by intensity (Figure S3).

### 2.5. Toxicity of GADD45α/GADD45β Amyloid-Like Aggregates

**Figure 6:**
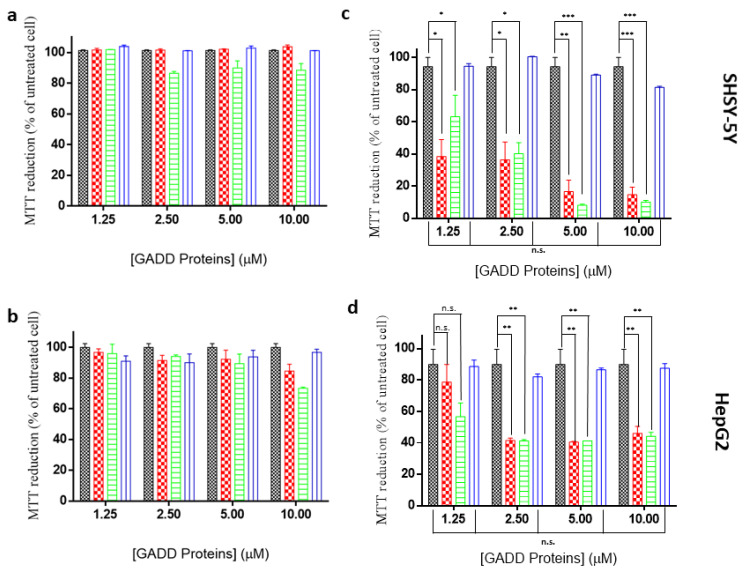
MMT viability assays performed on the SHSY-5Y (upper panels) and HepG2 (lower panels) cell lines after treatment with native GADD45 proteins (**a**,**b**) and thermally treated GADD45 proteins (**c**,**d**) at different concentrations. Black bars refer to the untreated cells; red bars refer to GADD45α; green bars refer to GADD45β; blue bars refer to GADD45γ. Statistical analyses are reported using Mann–Whitney T-test. * = *p*-value < 0.05; ** = *p*-value < 0.01; *** = *p*-value < 0.001. n.s. = not significative. Cell survival was expressed as percentage of viable cells in the presence of GADD proteins, compared to control cells grown in their absence. Error represent standard deviation. Experiments were repeated twice with similar results.

Since amyloid-like aggregates are frequently cytotoxic, we monitored the effects exerted by exposing human neuroblastoma (SHSY-5Y) and hepatoma (HepG2) cells to GADD45α/GADD45β aggregates and to the three proteins in the native states. Possible dose–response effects were evaluated at different protein concentrations (1.25, 2.5, 5, and 10 μM) monitoring cell viability at 24 and 48 h. As shown in Figure S4, none of the proteins exerted significant toxic effects on either SHSY-5Y or HepG2 cells after 24 h. After 48 h, a clear distinction between aggregated and non-aggregated forms emerged. Indeed, a high level of mortality was induced by the thermally treated forms of GADD45α/GADD45β, whereas no significant variation of the cell viability was produced by their native forms. On the other hand, neither native nor thermally treated GADD45γ, which does not form amyloid-like aggregates, induced significant alterations of cell viability (Figure 6).

## 3. Discussion

In its traditional version, the function–structure paradigm states that proteins acquire their functionality upon folding in a well-defined three-dimensional state. However, fundamental discoveries made in the last decade have progressively reshaped this concept. Indeed, it has been demonstrated that proteins frequently assume, in their functional form, an ensemble of states rather than a single structure. Moreover, it was found that significant portions of proteins are intrinsically disordered, and operate dynamically [19]. Finally, it has been shown that polypeptide chains have an intrinsic and unexpectedly strong propensity to self-assemble in misfolded states in which they assume a cross-β structure that is characteristic of amyloid aggregates [20,21]. In this scenario, here we performed a comparative biophysical characterization of the three members of the GADD45 protein family that are involved in a myriad of diversified biological processes. We found that, despite their remarkable sequence similarity, GADD45 proteins present rather distinct biochemical and biophysical properties. The analysis of the binding of these proteins to MKK7 indicates that, despite the general ability of recognizing the kinase by all members of the family, the affinity exhibited by GADD45α and GADD45γ is around one order of magnitude lower compared to that shown by GADD45β. This finding is in line with the observation that GADD45β binds the kinase through its loop 2 (residues 103–117), the region showing the most significant sequence variability among GADD proteins (Figure S2) [9]. Nevertheless, binding data suggest MKK7 as a potential biological partner also of GADD45α and GADD45γ. 

The findings emerged from the biophysical characterization of the proteins are particularly surprising as they demonstrate that GADD45β and GADD45γ are only marginally stable at physiological temperature, whereas GADD45α presents a Tm value expected for a protein isolated from a mesophilic organism. Even more intriguing are the structural properties of the species formed at high temperatures. While GADD45γ retains a significant level of secondary structure at high temperature and is able to significantly regain the original folding upon cooling, GADD45α and GADD45β when heated form aggregated species enriched in β-structure, despite the remarkable content of α-helix in their native structures. Moreover, the spectroscopic characterization of these aggregates clearly indicates that they possess amyloid-like features as they bind the dye ThT and present the characteristic intrinsic UV/blue fluorescence emission [22,23,24]. As found for many amyloid-like species, the aggregates formed by GADD45α and GADD45β present a remarkable toxicity against SHSY-5Y and HepG2 cells. 

The concomitant occurrence in GADD45β of a very limited thermal stability and of a remarkable propensity to form cytotoxic amyloid-like assemblies may appear a puzzling observation. Based on these observations, we hypothesize that the toxic species formed by GADD45β at physiological temperatures might represent a protection mechanism toward the pro-survival role played by this protein in several tumor tissues [25]. Similarly, the relative stability even at high temperatures of GADD45α, which has mostly pro-apoptotic features, might reflect a form of protection against undesired growth properties acquired by some cell types. Considering the frequent tendency of the polypeptide chains to undergo amyloid-like aggregation, the folding of proteins is no longer seen as mere mean to provide the correct spatial arrangement of residues for function and interaction but it might represent an effective way to promote or limit the formation of harmful species. In line with this concept, it was recently demonstrated—in a genomic-scale survey—that protein stability is an effective way to prevent aggregation [26]. Moreover, the extra stability of proteins isolated from mesophilic organisms has been linked to the presence in their sequences of amyloidogenic regions [27]. 

The rare propensity of GADD45β to form potentially cytotoxic species in near-physiological conditions may also be connected to some other functional features of the protein. In particular, it can be hypothesized that the observed propensity of GADD45β to establish biological partnerships and its limited expression levels may be effective ways to avoid the formation amyloid-like toxic assemblies. Alternatively, its tendency to form aggregates might represent a signal for its clearance when it is no longer needed or when its concentration exceeds harmful levels. Indeed, intracellular protein aggregates can be efficiently removed by a number of distinct mechanisms that include autophagy or secretion outside of the cells [28]. 

In conclusion, the low stability and/or the propensity to form toxic species, as here revealed though biophysical/biochemical studies might represent crucial properties of GADD45 proteins that must be considered in interpreting their many biological functions. The present finding also represent a stimulus for further investigations aimed at detecting the potential aggregation of these proteins in vivo. Therefore, the analysis of the GADD45β aggregation, which could also be modulated using inhibitors of amyloid-like aggregation [29,30,31], in cell model systems, overexpressing the protein, might represent a valuable mean to evaluate the biological consequences of this process. Moreover, since most proteins assemble into amyloid-like fibrils in vitro under extreme conditions, it has been pointed out that the study of the rare aggregation-prone species that form amyloids under physiologically relevant conditions might represent an important challenge that can provide interesting insights into protein aggregation [32]. In this scenario, further biophysical investigations on the GADD45α/GADD45βaggregation protein may provide remarkable results also in this field.

## 4. Materials and Methods

### 4.1. Cloning, Expression, and Purification of GADD45 Proteins

The genes of human GADD45α and GADD45γ were purchased from Sigma Aldrich and cloned into the pETM-13 expression vector using Bam-H1 and XhoI enzymes. Human GADD45β (hereafter only GADD45β) was cloned into a pET-28a+ vector as previously reported. GADD45α, GADD45β, and GADD45γ were expressed using *E. coli* BL21(DE3) cells, which were grown at 37 °C until 0.6–0.8 OD. Protein expression was induced by the addition of 0.5 mM isopropyl-D-thiogalactoside for 16 h at 22 °C. The cultures were harvested by centrifugation for 15 min at 4 °C and 6000 rpm. The pellets were re-suspended in lysis buffer (50 mM Tris-HCl pH 8, 500 mM NaCl, 5 mM DTT, 5% glycerol) and sonicated for 20 min (Misonix Sonicator 3000, Misonix Inc, NY, USA). Supernatants were harvested by centrifugation, for 30 min at 4 °C at 16,500 rpm. Soluble fractions were loaded on a Ni^2+^- NTA resin (Qiagen, Milano, Italy) previously equilibrated with lysis buffer. Proteins were eluted increasing the imidazole concentration. GADD45α, GADD45β, and GADD45γ were loaded onto a Superdex 200 16/60 column (GE Healthcare, Chicago, IL, USA) connected to a AKTA Purifier system (Fast Protein Liquid Chromatography, GE Healthcare, Chicago, IL, USA). The column was equilibrated in a buffer containing 20 mM Tris-HCl pH 7.5, 100 mM NaCl, 5 mM DTT, 5% glycerol. Yield and the purity of proteins were assessed by 15% SDS-PAGE analysis. Concentration was determined by the Bradford assay using BSA as reference. The recombinant kinase domain of human MKK7 (hereafter MKK7_KD) was obtained as His-fusion protein as previously reported [7,18,33].

### 4.2. Scattering (LS) and Dynamic Light Scattering (DLS) 

Light scattering measurements were performed using both a size exclusion chromatography coupled with light scattering (SEC-LS) and dynamic light scattering (DLS). SEC-LS was conducted using a semi-preparative SEC column (Superdex S200 10/30, GE Healthcare, Chicago, IL, USA) coupled to a light scattering detector (miniDAWN TREOS, Wyatt Technology Corporation, CA, USA) and to a differential refractive index detector (Shodex RI-101, Wyatt Technology Corporation, California, USA). The purification was conducted loading 0.5–1.0 mg of homogeneous sample on the column equilibrated in 20 mM Tris–HCl pH 7.5, 100 mM NaCl, 5 mM DTT, (*v*/*v*) 5% glycerol as running buffer, at a flow rate of 0.5 mL/min. Data were recorded and analyzed using the Astra software (version 5.3.4, Wyatt Technology Corporation, California, USA). Molecular size dispersion as a function of the hydrodynamic radius (Rh) and the aggregation status were determined by DLS. These experiments were performed at 25 °C using a Zetasizer Nano ZS (Malvern Instruments, Westborough, MA, USA) equipped with a 173° backscatter detector. Measurements were performed in triplicate using recombinant proteins at a concentration of 10 μM before and after thermal denaturation. Data were analyzed using the OMNISIZE software (Viscotek, Malvern Instruments, Westborough, MA, USA). 

### 4.3. CD Spectroscopy

Far-UV circular dichroism (CD) spectra of the proteins were recorded on a J-810 spectropolarimeter equipped with a Peltier temperature control system (Model PTC-423-S, Jasco Europe, Cremella, Italy). In all experiments, the protein concentration was 10 μM. Measurements were carried out in the 198–260 nm range at 20 °C using a 0.1 cm optical path length cell with the following parameters: 4 s as time constant, a 2 nm bandwidth, and a scan rate of 20 nm min^−1^. Three acquisitions for each spectrum were accumulated and averaged. Thermal denaturation curves were recorded at 222 nm between 20 °C and 100 °C, with a scan rate of 1 °C/min using tightly closed quartz cuvettes to prevent solvent evaporation. All data were expressed as molar ellipticity per residue (θ).

### 4.4. Bio-Layer Interferometry (BLI)

Bio-Layer Interferometry (BLI) was used to quantify the affinity and the selectivity of binding of the GADD45 proteins to MKK7_KD. The BLI experiments were carried out using an Octet^®^ RED96 System (AlfaTest) equipped with an array of eight independent probing BLI needles. Briefly, GADD45 proteins were dialyzed in 0.01 M HEPES pH 7.4, 0.15 M NaCl, 3 mM EDTA, 0.005% *v/v* Surfactant P20, 2 mM DTT (running buffer) and opportunely biotinylated using EZ-Link Sulfo-NHS-LC-Biotinylation Kit (Thermo Fisher, Waltham, MA, USA) following the manufacturer instructions. Subsequently, the biotinylated proteins were efficiently immobilized on the super streptavidin chip (SSA biosensor, ForteBio Pall) achieving a similar immobilization level on all sensors (2.5 nm). Dose–response assays were simultaneously carried out using MKK7_KD solutions at concentrations ranging between 9 and 300 nM, opportunely diluted in the running buffer. Data were analyzed using the Octet Data Analysis software and fitted by a 1:1 binding model.

### 4.5. Thioflavin T (ThT) Assay

Thioflavin T (ThT) fluorescence assays were carried out using a Varian Cary Eclipse spectrofluorometer (Varian, Palo Alto, CA, USA). The protein samples were placed in a quartz cell of 10 mm path-length at 10 μM concentration. A 440 nm radiation was used for the excitation while the emission was recorded in the interval 450–600 nm.

### 4.6. MTT Assays

Human SH-SY5Y neuroblastoma cells and human HepG2 hepatocellular carcinoma cells (American Type Culture Collection, Manassas, VA, USA) were grown at 37 °C in a humidified atmosphere with 5% CO_2_ in the Dulbecco’s modified medium (DMEM) supplemented with 10% fetal bovine serum, 1% glutamine, 100 U/mL penicillin, and 100 µg/mL streptomycin (EuroClone, Italy). For the MTT (3-(4,5-dimethylthiazol-2-yl)-2,5-diphenyltetrazolium bromide) assays 10^4^ SH-SY5Y and HepG2 cells were seeded in 96-well plates. Pre-heated GADD45α, GADD45β, and GADD45γ were added at 37 °C to the cells at different concentrations (1.25, 2.5, 5.0, and 10 µM) for either 24 or 48 h. Cell viability was assessed by the MTT reduction assay [34]. In brief, SH-SY5Y and HepG2 cells were washed with PBS and incubated at 37 °C for 4 h with a 0.5 mg/mL solution of MTT dissolved in DMEM. Subsequently, these cells were lysed with an isopropanol solution containing 10% (*v*/*v*) Triton-X100 and 8% (*v*/*v*) of a 1 M HCl solution. Absorbance of blue formazan was determined at 570 nm. Cell viability was expressed as the percentage of MTT reduction in treated cells compared to that observed in untreated cells.

## Supplementary Materials

The supplementary materials are available online at https://www.mdpi.com/article/10.3390/ijms221910700/s1.
