# Supplementary material for: Members of the GADD45 Protein Family Show Distinct Propensities to form Toxic Amyloid-Like Aggregates in Physiological Conditions
Source: Int J Mol Sci. 2021 Oct 2;22(19):10700. doi: 10.3390/ijms221910700 (PMC8509203; doi:10.3390/ijms221910700)
Supplement: Supplementary file 1 [file ijms-22-10700-s001.zip › ijms-1383005-SI.pdf]

Title Page

Members of the GADD45 protein family show distinct propensities to form toxic amyloid-like aggregates in physiological conditions

Authors

Giovanni Smaldone[1^], Daniela Caruso[2^], Annamaria Sandomenico[2], Emanuela Iaccarino[2], Annalia Focà[2], Alessia Ruggiero[2], Menotti Ruvo[2*], Luigi Vitagliano[2*]

Affiliations

[1] IRCCS SDN, Napoli, Via E. Gianturco 113, 80143 Napoli Italy

[2] Institute of Biostructures and Bioimaging, C.N.R, Via Mezzocannone 16, 80134, Napoli, Italy.

Email: luigi.vitagliano@unina.it; menotti.ruvo@unina.it

^Equally Contributed

*Corresponding authors

Keywords protein aggregation; amyloid-like toxicity, structure-stability relationships

Abbreviations

GADD, Growth Arrest and DNA Damage-inducible;

CD; Circular dichroism;

ThT; Thioflavin T

MKK7 Mitogen-Activated Protein Kinase 7

MKK7_KD Mitogen-Activated Protein Kinase 7_ Kinase Domain

BLI Bio-Layer Interferometry

Highlights – (three to five)

GADD45α, GADD45β, and GADD45γ presents distinct thermal stabilities and unfolding pathways

GADD45α and GADD45β form amyloid-like aggregates upon thermal treatments

GADD45α and GADD45β aggregates are toxic against SHSY-5Y and HepG2 cells

GADD45β forms toxic aggregates in nearly physiological conditions

Present findings provide a new framework for interpreting the many functions of GADD45 proteins

A

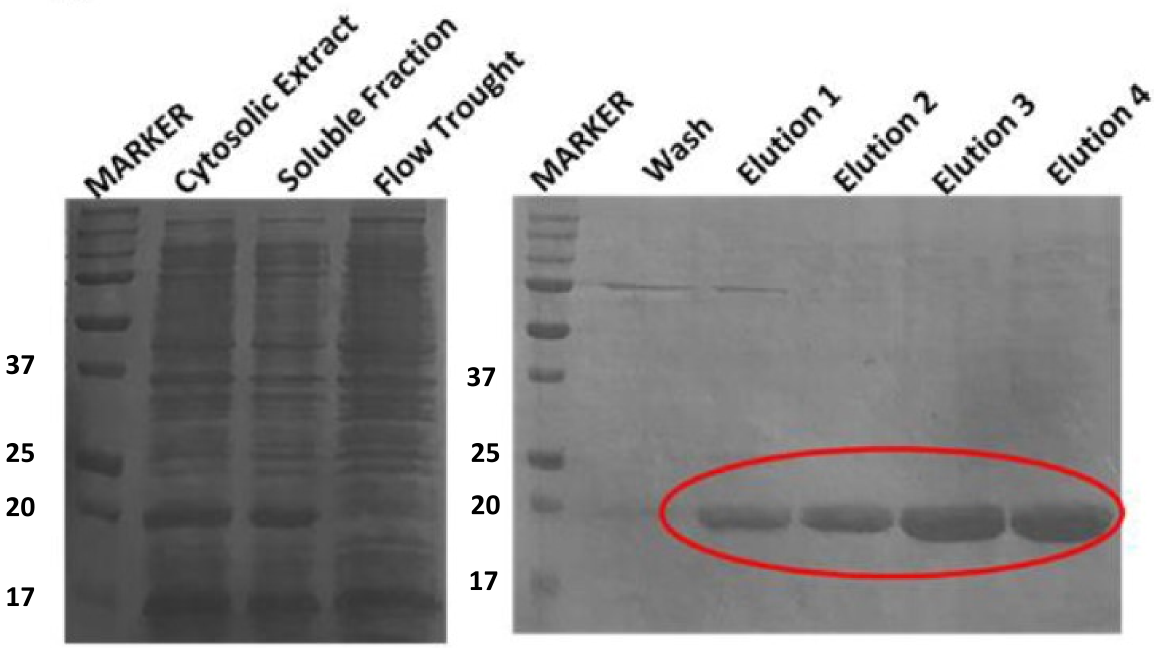

B

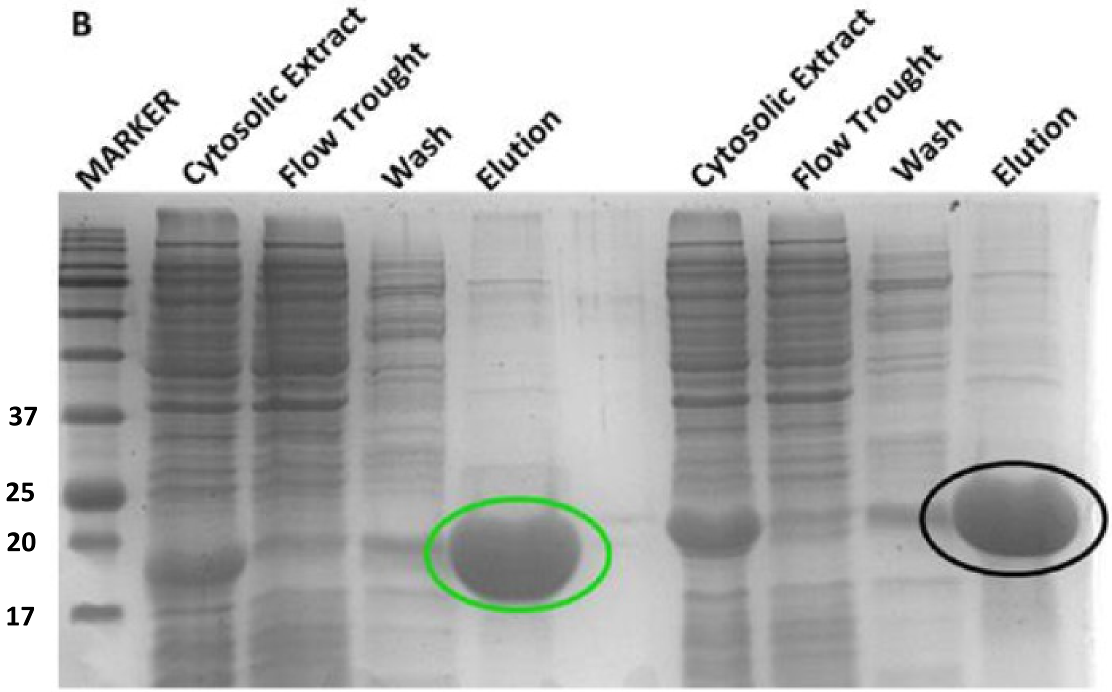

Figure S1. *SDS-PAGE analysis of GADDβ (A), GADDα and GADDγ (B) Hys-Trap purification. Numbers represent molecular weight of protein marker expressed in kDA.*

```
GADDγ           MTLEEVRGQDTVPESTARMQGAGKALHELLLSAQRQGCLTAGVYESAKVLNVDPDNVTFC 60
GADDα           MTLEEFSAGE---QKTERMDKVGDALEEVLSKALSQRTITVGVYEAAKLLNVDPDNVVLC 57
GADDβ           MTLEELVACD---NAAQKMQTVTAAVEELLVAAQRQDRLTVGVYESAKLMNVDPDSVVLC 57
                *****. . :   : : :*: .  *:.*:*  *  *  :*.****:**::*****.*.:*

GADDγ           VLAAGEEDEGDIALQIHFTLIQAFCCENDIDIVRVGDVQRLAAIVG--------AGEEAG 112
GADDα           LLAADEDDDRDVALQIHFTLIQAFCCENDINILRVSNPGRLAELLLLETDAGPAASEGAE 117
GADDβ           LLAIDEEEEDDIALQIHFTLIQSFCCDNDINIVRVSGMQRLAQLL-----GEPAETQGTT 112
                :** .*::: *:**********:***:***:*:**..  *** ::           : :

GADDγ           APGDLHCILISNPNEDAWKDPALEKLSLFCEESRSVNDWVPSITLPE-     159
GADDα           QPPDLHCVLVTNPHSSQWKDPALSQLICFCRESRYMDQWVPVINLPER     165
GADDβ           EARDLHCLLVTNPHTDAWKSHGLVEVASYCEESRGNNQWVPYISLQER     160
                   ****:*::**: . **. .* ::  :*.***  ::*** *.* *
```

Figure S2. Multiple alignment of GADDα, GADDβ and GADDγ sequences. Symbol code: *= conserved residues, (. :) non conserved residues sharing high (:) or moderate (.) chemico-physical properties. Color code: hydrophobic residues – red; positively charged residues – pink; negatively charged residues – blue; polar residues – green;

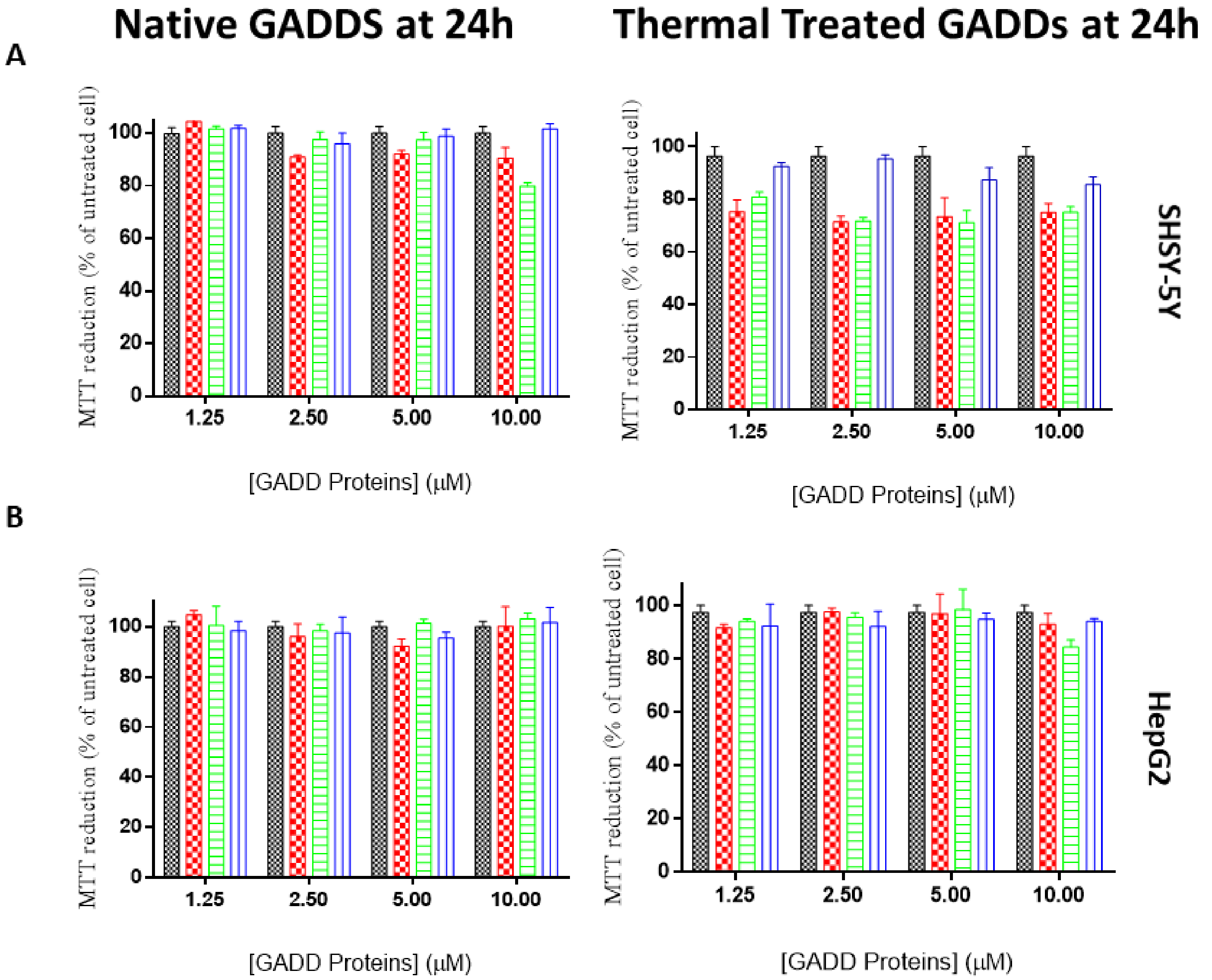

Figure S3. MTT assay on SHSY-5Y and HepG2 cell lines at 24h of incubation. MMT viability assays performed on the SHSY-5Y (A) and HepG2 (B) cell lines after treatment with native GADD45 proteins (left) and thermal treated GADD45 proteins (right) at different concentrations. Black bars refer to the untreated cells; red bars refer to GADD45α, green bars refer to GADD45β; blue bars refer to GADD45γ. No significative differences occurs in every conditions tested. Cell survival was expressed as percentage of viable cells in the presence of GADD proteins, compared to control cells grown in their absence. Error represent Standard Deviation. Experiments were repeated twice with similar results.

Table S1. Theoretical versus experimental molecular weights of the three GADD45 proteins Molecular weights consider the presence of different His-Tag in the three isoforms. The experimental molecular weights are in close agreement (within few Da) if the loss of the N-terminal methionine (Δ molecular mass = -131.2) is assumed .

PROTEIN	THEORETICAL MOLECULAR MASS (Da)	EXPERIMENTAL MOLECULAR MASS (Da)
**GADD45α**	20499.1[a]	20367.9[a]
**GADD45β**	19327.7[b]	19192.1[b]
**GADD45$_{\gamma}$**	19284.6[a]	19153.6[a]

[a]The construct contains an N-terminal His-tag :**MGSSHHHHHH SSGLVPRGSH**

[b]The construct contains an N-terminal His-Tag: **MGSSHHHHHH GGIP**
